# Preliminary Efficacy and Feasibility of “Thinking While Moving in English”: A Program with Physical Activity Integrated into Primary School English Lessons

**DOI:** 10.3390/children5080109

**Published:** 2018-08-10

**Authors:** Myrto F. Mavilidi, David R. Lubans, Narelle Eather, Philip J. Morgan, Nicholas Riley

**Affiliations:** Priority Research Centre for Physical Activity and Nutrition, School of Education, University of Newcastle, Callaghan, NSW 2308, Australia; David.Lubans@newcastle.edu.au (D.R.L.); Narelle.Eather@newcastle.edu.au (N.E.); Philip.Morgan@newcastle.edu.au (P.J.M.); Nicholas.Riley@newcastle.edu.au (N.R.)

**Keywords:** learning, primary school children, academic achievement, on-task behavior, cognition, physical activity

## Abstract

*Background*: The physical, cognitive, and learning benefits of physical activity for children have already been established. However, many schools are failing to provide children with sufficient activity at school due to a crowded school curriculum. Physical activity interventions that integrate physical activity with learning is a way to enhance physical and cognitive benefits without loss of academic time. This study evaluated the preliminary efficacy and feasibility of “Thinking While Moving in English”, a primary school program that integrates physical activity into English lessons. *Method*: Two classes of Grade 4 students (*n* = 55, 10–11 years old) were randomly assigned to the intervention (*n* = 29) or control (*n* = 26) conditions. The program components consisted of 3 × 40 min physically active academic lessons per week, delivered over a 4-week period. The following measures were taken at baseline and immediate post-intervention: on-task behavior, cognition (inhibition and working memory), and learning outcomes (spelling and grammar). *Results*: Results revealed significant improvements in on-task behavior and spelling in the intervention group, compared to the control group. There were no observed improvements in cognitive outcomes or grammar. *Conclusions*: This study provides preliminary evidence for the efficacy of physically active English lessons to enhance children’s educational outcomes.

## 1. Introduction

Emerging evidence from cross-sectional studies suggests that physical activity is positively associated with children’s cognitive functioning and academic performance [1,2]. Nevertheless, the benefits of physical activity for young people’s psychological health (e.g., decreased anxiety and depression, psychological well-being) as well as physiological health (e.g., reduced risk of obesity and type II diabetes, improved physical fitness) are well established [3,4,5,6]. Despite this, the majority of young people are not sufficiently active and physical inactivity is rated as the fourth leading risk factor for mortality, globally [7].

Schools are well placed to address the global inactivity pandemic as they possess the necessary facilities, equipment and personnel required to engage children in a range of physical activities during a school day [8,9]. A multi-component approach is recommended for promoting physical activity in schools [10]. This is commonly defined as a collaboration between school staff, families and community members in providing a coordinated approach to delivering high-quality physical education, and implementing physical activity opportunities before, during, and after school. Despite the potential of school-based physical activity, interventions are rarely implemented as intended, and lack of time is the most commonly cited barrier by teachers [11].

With adequate training and support, teachers can successfully increase students’ opportunities for physical activity participation within a school day by using the following: (I) physically active classroom breaks, (II) curriculum-focused physical activity breaks, and (III) physically active academic lessons. The physically active classroom breaks are short bouts of physical activity performed as a break from academic instruction time (also referred to as activity bursts [12]), whereas the curriculum-focused physical activity breaks contain short bouts of physical activity that include curriculum content [13]. Finally, physically active academic lessons involve the integration of physical activity into academic lessons in key learning areas other than physical education [14,15]. Among the three types of classroom-based physical activity programs described, physically active academic lessons have been studied the least.

The main difference between curriculum-focused physical activity breaks and physically active academic lessons is duration. Curriculum-focused activity breaks typically involve short bouts of physical activity (i.e., 3–10 min) performed during a lesson that is usually aligned with the lesson content. For example, during a mathematics lesson, a teacher might provide students with an opportunity to practice multiplication tables while performing aerobic activity (e.g., running on the spot). Alternatively, physically active academic lessons require teachers to integrate physical activity specifically within the lesson content to enhance and reinforce learning. Physically active academic lessons are consistent with theories of embodied learning, which advocate the inter-relatedness of action and perception, emphasizing the role of sensorimotor experiences on perception, understanding, and learning [16,17]. For example, a mathematics lesson focused on measurement might involve students trying to create 2D shapes with set perimeters. As such, students could be given the task of making an irregular pentagon with a perimeter of 25 m using 5 markers. They would then travel around the perimeter before measuring with a trundle wheel. Traditionally students measure pre-drawn shapes using a ruler at their desk. Both achieve the same learning goal.

In general, teachers’ perceptions regarding movement integration in elementary schools are usually linked with their own attitudes towards physical activity, and are related to significant barriers and challenges, such as limited time to meet academic outcomes and respond to the overcrowded curriculum, lack of social and financial support from schools, resources, teacher training and experience [18,19,20]. Previous studies have demonstrated that physically active academic lessons can increase students’ on-task behavior, academic performance, and physical activity levels [21,22,23,24]. In addition, they are perceived by students as more enjoyable than traditional sedentary lessons [25,26,27]. For example, Mullender-Wijnsma et al. (2016) [24] implemented physical activity in mathematics and spelling over a 2-year study period using a 22-week (3 × per week) intervention in second and third grade students. The intervention group performed better than the control group on mathematics speed (*p* < 0.001, *ES* = 0.45), general mathematics (*p* < 0.001, *ES* = 0.42), and spelling (*p* < 0.001, *ES* = 0.45), but no benefits were found for reading. Similarly, Riley and colleagues’ EASY Minds program [15] evaluated the effects of 3 × 60 min physically active mathematics lessons in 8 primary schools (Grades 5 and 6) and found increased children’s physical activity levels (*p* = 0.008) and improved on-task behavior (*p* = 0.011).

Overall, the majority of studies have tested the effects of physically active mathematics, while less is known about integrating movement into English lessons. The current curriculum recommendations in New South Wales, Australia, require primary school students to spend 25–35% of a school week in English lessons, 20% in mathematics and only 6–10% in Personal Development, Health and Physical Education (PDHPE) [28]. Taking into account that the majority of English lessons are traditionally sedentary, replacing sitting time with interactive game-based and movement based lessons may have both physical and cognitive benefits. Importantly, executive functions are considered crucial for physical, emotional, psychological, and social development in children and have been associated with academic success [2,29]. Assessing children’s executive functions will allow us to infer on whether physically active lessons have the potential to modify both cognitive and academic outcomes.

Therefore, the aim of the study was to examine the preliminary efficacy and feasibility of the Thinking While Moving in English (TWM-E) program. The TWM-E program is a 4-week primary school curriculum-based intervention and the current study will aim to answer the following research questions:

- What is the impact of the TWM-E program on students’ on-task behavior, cognition and academic achievement?
- Is the TWM-E program feasible for delivery in primary schools?
- What were children’s perceptions of the TWM-E program?

## 2. Materials and Methods

### 2.1. Design

Study approval was sought and obtained from the University Research Ethics Committee (No: H-2017-0240), and the NSW Department of Education for governmental schools (SERAP, No. 2017368). This study examined the effects of the TWM-E intervention on on-task behavior, academic achievement, and cognitive outcomes. Hence, a mixed 2 × 2 between subjects-experimental design was used, comparing the experimental conditions (TWM-E vs. control), measured in two time points (baseline vs. post-test). The design, conduct and reporting of the TWM-E program adheres to the Consolidation Standards of Reporting Trials (CONSORT) guidelines [30].

### 2.2. Participants

This feasibility trial involved 55 Grade 4 students with a mean age of 10.26 (SD = 0.35) years from two classes of one primary school, who were randomly assigned to the control (*n* = 26) or TWM-E (*n* = 29) conditions. Each class was assigned to one condition. The demographic characteristics of participants are presented in Table 1. The majority of the participants were identified as having an Australian cultural background (70.9%), and having English as the spoken language at home (87.3%).

### 2.3. Procedure

TWM-E uses an innovative instructional approach, which integrates physical activity into English lessons. The advantage of TWM-E over school-based physical activity interventions is the use of the existing English curriculum in learning activity design, which enables teachers to meet subject syllabus requirements and physical activity outcomes simultaneously. The program ran for 4-weeks with 3 × 40 min lessons per week. The TWM-E lessons were delivered in the morning session (9:00–10:30 a.m.) during the usual scheduled English lessons. The TWM-E lessons were performed outside of the classroom, whereas the control group remained at their class for their lessons, which was the regular classroom routine.

### 2.4. Experimental Conditions

TWM-E condition: Children in this condition attended English lessons, taught by a member of the research team who is a qualified primary and physical education teacher with 25 years’ teaching experience. The lessons were designed in a way that movement was integrated into learning experiences from the Primary School English curriculum, based on the NSW Board of Studies syllabi. The lessons focused on spelling, grammar and phonemic awareness (see Table 2 for example of activities). For example, students were jumping or hopping on randomly placed-letters within a 3 × 3 square design drawn with chalk while spelling words. The lessons were conducted outside of the classroom. Movements were used to support and reinforce English concepts during academic instruction.

Control condition: Children in this condition followed the usual instruction without any modifications of the curriculum delivered by the classroom teacher inside the class.

### 2.5. Measures

Before the beginning of the intervention, baseline data were collected for on-task behavior, academic achievement, and cognitive measures. Firstly, the English lessons of the two classes (9:00–10:30 am) were observed, followed by the cognitive measures. Cognitive assessments were conducted individually, while assessments for academic achievement and on-task behavior were conducted as a group. These assessments were conducted by trained members of the research team. The academic achievement assessments were administered by teachers after the completion of the other assessments, to avoid overloading students. Identical procedures and materials were used for the post-test assessments. All assessments occurred during normal class time.

### 2.6. On-Task Behaviour

Classroom behavior was observed using momentary time sampling [31]. This observational tool has been adapted from the “Behavior observation of students in schools” [31] and the “Applied behavior analysis for teachers” [32]. On-task behavior refers to the time a child is actively engaged in an academic activity (e.g., reading, writing, or performing the designated task), rather than passively engaged (i.e., sitting quietly, but not engaged in the activity) or disruptive. Off-task behavior is related to behavior not associated with the task (e.g., off-task motor, walking around the class, off-task verbal, talking, or off-task passive, staring in the class [13,15]. Twelve students per class (6 males, 6 females) were randomly selected using a random number-producing algorithm. All students were observed by a member of the research team and a research assistant in 15-sec intervals on a rotational basis over a 30-min period in the allocated English time slot (9:30–10:30 am). Twelve observations per class at each time point (baseline and post-test) were included. A 2-h training focusing on identifying and classifying behavior into the appropriate categories, and a trial practice was provided to observers. Students were not aware of being observed at any given time, even though they noticed the presence of the research team in the class. Following all observations, the observers compared notes to clarify discrepancies. Classroom behavior was reported as a percentage of time categorized as “on-task” (consisted of “actively engaged” or “passively engaged”) and “off-task”.

### 2.7. Academic Achievement

Children’s spelling and grammar skills were assessed using the South Australian Spelling test and Grammar and Punctuation test. In the South Australian Spelling test, children were asked to write down a list of words called aloud by the teacher. This test can identify children’s spelling sub-skills from the number of errors [33]. The Grammar and Punctuation test was a teacher-made test (available online at https://www.twinkl.co.uk/search), in which children were asked to match words from the same family, use conjunctions, and punctuate direct speech and sentences. The teachers of each class administered the tests for approximately 20 min per test at both time points.

### 2.8. Cognitive Outcomes

Two measures of executive function (i.e., inhibition and working memory) were assessed through an online computer program: The Eriksen Flanker task is one example of stimulus evaluation tasks to measure inhibition. The Flanker test is an interference task, in which different inputs compete with the target. Participants are required to discriminate between arrows that have different direction. Congruent stimuli (e.g., →→→→→) elicit faster and more accurate responses, whereas incongruent stimuli (e.g., →→←→→) can slow down response speed and accuracy [34,35]. Participants were asked to indicate the direction of the center arrow as fast as they can. Their answers were recorded: the percentage number of correct answers (accuracy), the reaction time to complete the congruent tasks, as well as the time to complete the incongruent tasks (in seconds) were gauged.

Working memory was measured using a version (2-back) of the “n-back task”. Subjects are asked to monitor the identity or location of a series of nonverbal stimuli (i.e., pictures of ordinary objects such as cat or book) and indicate which presented stimulus are the same with the ones presented previously. The n-back working memory paradigm has been shown to be a powerful tool measuring process and content-specific activation of working memory [36]. Participants’ answers were recorded: the percentage of correct answers (accuracy) and the reaction time to complete the tasks (in seconds) were gauged. The cognitive assessments lasted approximately 15 min per child at both time points.

### 2.9. Process Evaluation

At the completion of the intervention, children in the intervention condition completed a short evaluation questionnaire (see Table 3), which included items assessing opinions of the overall program and appropriateness of program content (4 items), instructor quality (4 items), timing (2 items), and program impact on children’s perceptions about physical activity (8 items). The questions used a 5 point Likert scale ranging from 1 (strongly disagree) to 5 (strongly agree).

As part of the process evaluation, a record was made of students’ overall physical activity during the TWM-E lessons. During the learning sessions, children’s physical activity was measured using pedometers (Yamax Digi walker sw700), which were clipped around the waist and positioned over the anterior aspect of the right hip. The pedometers were worn from the beginning of each 40 min TWM-E lesson and were removed at the end. Data were reported as steps, calculated by the average number of steps per week. Pedometers have been shown to accurately capture changes in physical activity in children [37,38].

### 2.10. Statistical Analysis

Analyses of the outcomes were conducted using linear mixed models in IBM SPSS Statistics, version 23.0 (2010 SPSS Inc., IBM Company, Armonk, NY, USA). Alpha levels were set at *p* < 0.05. The models were used to assess the impact of the intervention (control or TWM-E), time (treated as categorical with levels baseline and 4-weeks), and the group-by-time interaction, using a random intercept to account for the repeated measures of each participant. Cohen’s d was also calculated and interpreted as follows: *d* = 0.2, ‘small’ effect size; *d* = 0.5, ‘medium’ effect size; and *d* = 0.8, ‘large’ effect size [39]. A summary of the outcome measures is demonstrated in Table 4 (at baseline, and 4-weeks, adjusted mean differences and effect sizes).

## 3. Results

### 3.1. Preliminary Analyses

Baseline comparisons showed that there were no significant differences between the two conditions in the baseline measures of spelling (t(49) = 1.34, *p* = 0.187), grammar (t(35.24) = −1.34, *p* = 0.190), accuracy in the inhibition task (t(53) = −0.99, *p* = 0.325), reaction time in congruent inhibition tasks (t(53) = −0.23, *p* = 0.816), and incongruent tasks (t(53) = −0.94, *p* = 0.350), accuracy in the working memory task (t(53) = 0.59, *p* = 0.561), reaction time in the working memory task (t(53) = −0.02, *p* = 0.987). There were significant differences between the two conditions in children’s active engagement (t(22) = −2.5, *p* = 0.020), and off-task behavior (t(22) = 4.36, *p* ≤ 0.001), with worse performance shown in the TWM-E condition. No differences found in children’s passive engagement (t(22) = −1.46, *p* = 0.160).

### 3.2. Main Analyses

#### 3.2.1. On-Task Behavior

Significant group-by-time effects were observed for children’s active engagement in favor of the TWM-E group [adjusted mean difference = 58.33 (95% CI, 43.51 to 73.16), *p* ≤ 0.001, *d* = 1.7]. No significant group-by-time effect was observed for children’s passive engagement [adjusted mean difference = −9.17 (95% CI, −22.97 to 4.63), *p* = 0.182]. Finally, significant group-by-time effects were observed for off-task behavior, favoring the TWM-E group [adjusted mean difference = −49.58 (95% CI, −63.81 to −35.35), *p* ≤ 0.001, *d* = −1.6].

#### 3.2.2. Academic Achievement

Significant group-by-time effects were observed for spelling scores [adjusted mean difference = 3.60 (95% CI, 0.59 to 6.62), *p* = 0.020, *d* = 0.07]. The effect sizes were in favor of the TWM-E group. Non-significant group-by-time effects were observed for grammar scores.

#### 3.2.3. Cognitive Outcomes

There were no significant group-by-time effects for inhibition and working memory.

#### 3.2.4. Process Evaluation

Students from the intervention group reported that the overall program was enjoyable (*M* = 4.19, *SD* = 0.86), the teacher was understanding and delivered fun activities (*M* = 4.10, *SD* = 0.97), the program timing was appropriate (*M* = 3.83, *SD* = 0.86), and the program had an overall positive impact on children’s perceptions about physical activity (*M* = 3.40, *SD* = 1.16).

In terms of physical activity, group effects were observed for physical activity [F(1,50) = 111.75, *p* ≤ 0.001; TWM-E *M* steps = 1534.60, *SD* = 74.75; control *M* steps = 371.45, *SD* = 80.74].

## 4. Discussion

The primary aim of this study was to assess the preliminary efficacy and feasibility of a movement-based English program in primary schools. The Thinking While Moving in English (TWM-E) intervention resulted in significant intervention effects for on-task behavior, and academic achievement in spelling. The TWM-E program was well-received and enjoyed by students and teachers.

The significant improvements for children’s on-task behavior and reduced off-task behavior align with previous research showing that school-based physical activity programs can positively influence on-task behavior [13,15]. The large effect sizes can confirm the effectiveness of the intervention on on-task behavior, which can also predict academic success [40]. Specifically, post-test, children’s spelling skills in the TWM-E group were significantly higher than those in the control condition. However, this was not the case for the grammar test, in which no group-by-time effects were found. A more sensitive measure or an extended study period may be needed to detect changes or improvements in grammar.

Overall, the current study replicates the results of previous literature on academic performance that integrates physical activity across different learning domains [14,15,25,26,27,41]. This innovative instructional approach lays the foundation for the purposeful engagement of the motor system in learning. The use of language, body, and interaction with the external environment can be used as adjunct during the learning process [42]. The inclusion of movements that are cognitively engaging contribute to the construction of conceptual representations, which are based on tangible and concrete information. Thus, they leave a deeper and stronger memory trace, and are more easily retrieved by students [43].

Concomitantly, existing literature supports the positive impact of physical activity on cognitive and academic performance [2,44,45]. A recent systematic review suggests a range of potential neurobiological (e.g., improved blood flow and oxygenation, synaptic plasticity), psychosocial (e.g., positive physical self-perceptions, mood and emotions, and social connectedness), and behavioral mechanisms (e.g., improved sleep volume and quality, coping and self-regulation skills) that may explain the positive effect of physical activity on academic outcomes [6]. Of note, improvements in children’s and youth’s cognitive functioning have been shown after acute and repeated (i.e., chronic) bouts of exercise [46,47,48]. Exercise of sufficient intensity and duration to improve cardiorespiratory fitness may improve cognitive performance via a range of neurobiological mechanisms [49,50]. Alternatively, cognitively engaging physical activity of varying intensity may have benefits for young people via behavioral (e.g., on-task behavior in the classroom) and psychosocial (e.g., motivation) mechanisms. In the current study, the intensity of physical activity was not measured. It could be argued though, that the intensity and duration of physical activity was possibly not sufficient to provoke neurobiological changes. Children’s cognitive scores for inhibition followed a non-significant positive trend direction, regardless of the group, with an increase of accuracy and decrease of reaction times from baseline to post-test. However, no group-by-time effects were found for working memory. Normative data for children between 7–13 years old showed that age is a strong predictor of the n-back task [51]. At age 7, children can have 66% success rates at the 1-back task, while only 37% at the 2-back task. Previous literature report that the 1-back task can be completed by children 10–12 years, and the 2-back task can be improved throughout adolescence [52,53].

Finally, the measure of physical activity demonstrated that on average children in the TWM-E condition performed more steps per English lesson during the study period than the control group for the 4-weeks of program implementation. Our findings support that school-based physical activity interventions are feasible to implement and may contribute to the activity accumulated by young people at school [54,55]. Previous research with physically active mathematics lessons has shown significant increases in children’s physical activity levels [15]. After covering the curriculum content, a dual goal is set with combined physical and cognitive improvements. Considering the mental and physical health benefits of physical activity in children and youth [6], the stealth interventions that promote physical activity can achieve the desirable outcomes on increasing children’s physical activity levels [56].

Although the results of this study are promising, some limitations need to be noted. Firstly, the small sample size of the study makes it difficult to generalize the results. Second, the intervention was delivered by a member of the research team within the school context. Training teachers and providing them with professional learning development on how to integrate physical activity within their lessons plans would ensure a higher ecological validity, and would give them more flexibility to adjust the lessons based on their needs. In addition, standardized assessments for grammar and punctuation might be more insightful. Moreover, this study measured physical activity for a very brief time as part of process evaluation only. Possibly, there are potential compensation effects in subsequent activity in the day that cannot be detected by looking at in-class physical activity only. Future studies can include baseline measurements using more accurate tools for physical activity (e.g., accelerometers [57]). Finally, the study was conducted over a 4-week period. Previous studies have demonstrated that longer time periods may be needed to elicit improvements in cognitive functions [50].

## 5. Conclusions

The Thinking While Moving in English program was successful in improving on-task behavior and spelling scores in primary school children. The program successfully integrated physical activity into the existing English curriculum, providing a feasible strategy for meeting both academic and physical activity outcomes within the current school context. These findings highlight the potential of school-based physical activity interventions (i.e., physically active lessons) on improving learning outcomes and increasing physical activity [58]. Based on Beets et colleagues [59], in order to increase children’s physical activity levels, it is important to expand, enhance, or extend. This study can be considered as an example of expanding, as physical activity is not typically delivered during English lessons. This successful feasibility trial will be used to inform a larger clustered randomized controlled trial and provide further evidence of program effectiveness and sustainability.

## Figures and Tables

**Table 1:** Participant demographic characteristics.

Characteristics	Control (*n* = 26)	TWM-E (*n* = 29)	Total (*n* = 55)	*p* Value
Age (years), mean (SD)	10.29 (0.42)	10.22 (0.27)	10.26 (0.35)	0.433 ^a^
Sex, *n* (%)				0.875 ^b^
Male	14 (53.8)	15 (51.7)	29 (52.7)	
Female	12 (46.2)	14 (48.3)	26 (47.3)	
Cultural background, *n* (%)				0.602 ^b^
Australian	18 (69.2)	21 (72.4)	39 (70.9)	
European	2 (7.7)	4 (13.8)	6 (10.9)	
Asian	4 (15.4)	2 (6.9)	6 (10.9)	
Other	2 (7.7)	2 (6.9)	4 (7.3)	
Language spoken at home, *n* (%)				0.576 ^b^
English	22 (84.6)	26(89.7)	48 (87.3)	
Other	4 (15.4)	3(10.3)	7 (12.7)	
Aboriginal or Torres Strait Islander, *n* (%)				
Yes	26 (100)	29 (100)	55 (100)	
No	0 (0)	0 (0)	0(0)	

^a^ Independent *t*-test, ^b^ χ^2^ test.

**Table 2:** Examples of movement-based learning in English.

Academic Concepts	Description of Activities
Spelling	*Activity 1: Spelling fitness* Students in pairs try to recall and spell out loud their weekly spelling words whilst either skipping with a rope, performing squats, push-ups or any exercise of their choice. Partner checks provides feedback and students swap roles. *Activity 2: Basketball dribble* Lettered flexi domes are placed randomly on a playground area. Students in pairs check their spelling word chosen at random by their partner and dribble around the letters to complete the spelling. Partner checks and times if students choose. *Activity 3. Hopscotch* A square is drawn in chalk on the playground. (3 m by 3 m). This is then divided into 9 equal squares. Students or Teachers then choose a nine letter word e.g., telegraph. Students then either hop or jump from square to square when they see a word and record this, e.g., graph, late, page etc. Students rotate between several squares every few minutes. Extension: same activity with bean bags containing vowel digraphs and students record words e.g., ea, ou, ie, etc. *Activity 4 Homophone hurry* Students are given sentences with a missing word, e.g., Did they get ______ magazine yet? Students decide in the missing word and complete the correct action, e.g., there (10 push-ups), their (10 squats) they’re (1o repetitions of their choice).
Grammar+	*Activity 1: Rob the nest* Bean bags with letters are placed in the middle of a square. Students are in groups of or 4 (max) in the four corners. Students run in and collect 1 bean bag and high 5 team mate who continues etc. On a whistle, students stop robbing the nest and have to record an adjective, noun and verb beginning with each letter. *Activity 2: Active adverbs* Students in pairs look at a list of sentences containing adverbs, e.g., He quickly ran down the road to buy milk. Students record the adverb and travel across the playground and back using the following FMS depending on if the adverb describes how: side gallop, where: skip or when: shuttle run. *Activity 3: Athletic apostrophes* Students are given a list of words e.g., “You are”, and they have to contact the words and then perform movements whilst saying the letters. For example, for “you’re” students would go y (squat), o (squat), u (squat) apostrophe (jump) r (squat) e (squat).

**Table 3:** Program evaluation questionnaire.

Program Evaluation Questionnaire	*M* (*SD*)	*N*
*Program*		
The program was enjoyable.	4.04 (0.96)	26
I looked forward to the lessons.	3.92 (1.09)	26
I liked being physically active in the sessions.	4.35 (0.75)	26
I liked working outside the classroom.	4.46 (0.65)	26
*Instructors* (*Research Team*)		
I liked having the instructor deliver the program.	4.04 (1.04)	26
The instructor made the activities fun.	4.15 (1.05)	26
The instructor was enthusiastic.	4.08 (0.98)	26
The instructor made the activities easy to understand.	4.12 (0.81)	26
*Timing*		
The program length (4 weeks) was good.	3.96 (0.87)	26
The number of sessions (3/week) was right.	3.69 (0.84)	26
*Impact*		
After participating in Thinking While Moving in English I have more positive feelings about physical activity.	3.81 (0.98)	26
After participating in Thinking While Moving in English I feel better about myself.	3.27 (0.96)	26
After participating in Thinking While Moving in English at Home I find it easier to concentrate in class.	3.04 (1.15)	26
After participating in Thinking While Moving in English I am more active.	3.50 (1.21)	26
I enjoyed participating in the Thinking While Moving in English program.	3.92 (1.13)	26
I now feel more confident in doing my English work.	2.85 (1.19)	26
The program has encouraged me to be more physically active.	3.50 (1.33)	26
My involvement in the program has increased my knowledge of the importance of regular physical activity.	3.31 (1.35)	26

Note: 1 = Strongly Disagree, 2 = Disagree, 3 = Neutral, 4 = Agree, 5 = Strongly Agree.

**Table 4:** Summary of outcome measures.

Outcomes	Group	Baseline	4-weeks	Time *p*	Adj. Diff in Change	Group-by-Time *p*	Group-by-Time *d*
**On-task behavior**							
Actively engaged	CON	48.33 (37.60 to 59.06)	35.42 (28.15 to 42.69)	0.018	58.33 (43.51 to 73.16)	<0.001	1.7
	TWM-E	30.00 (19.27 to 40.73)	75.42 (68.15 to 82.69)	<0.001			
Passively engaged	CON	39.17 (31.61 to 46.73)	36.25 (29.64 to 42.86)	0.542	−9.17 (−22.97 to 4.63)	0.182	0.6
	TWM-E	31.67 (24.11 to 39.23)	19.58 (12.98 to 26.19)	0.018			
Off-task behavior	CON	12.50 (3.67 to 21.33)	27.92 (22.36 to 33.47)	0.004	−49.58 (−63.81 to −35.35)	<0.001	−1.6
	TWM-E	38.75 (29.92 to 47.58)	4.58 (−0.97 to 10.14)	<0.001			
**Academic achievement**							
Spelling	CON	48.73 (45.83 to 51.62)	48.22 (44.54 to 51.90)	0.638	3.60 (0.59 to 6.62)	0.020	0.7
	TWM-E	45.21 (42.44 to 47.98)	48.30 (44.83 to 51.78)	0.005			
Grammar	CON	6.12 (5.34 to 6.89)	6.24 (5.49 to 7.00)	0.714	−0.25 (−1.23 to 0.74)	0.617	0.2
	TWM-E	6.79 (6.05 to 7.55)	6.68 (5.95 to 7.41)	0.733			
**Cognitive function**							
Inhibition (accuracy)	CON	1.97 (1.96 to 1.99)	1.99 (1.99 to 2.00)	0.016	−0.01 (−0.04 to 0.13)	0.330 *	0.1
	TWM-E	1.98 (1.97 to 2.00)	1.99 (1.99 to 2.00)	0.234			
Inhibition (reaction time for congruent tasks)	CON	3.01 (2.93 to 3.09)	2.93 (2.88 to 2.98)	0.026	0.04 (−0.06 to 0.14)	0.414 *	0.1
	TWM-E	3.01 (2.93 to 3.08)	2.96 (2.92 to 3.01)	0.227			
Inhibition (reaction time for incongruent tasks)	CON	3.18 (3.09 to 3.27)	2.97 (2.92 to 3.03)	<0.001	0.017 (−0.09 to 0.13)	0.762 *	0.2
	TWM-E	3.21 (3.13 to 3.30)	3.03 (2.97 to 3.08)	<0.001			
Working memory (accuracy)	CON	44.83 (36.93 to 52.72)	42.14 (36.53 to 47.76)	0.588	9.73 (−3.87 to 23.33)	0.157	0.0
	TWM-E	41.66 (34.19 to 49.14)	48.70 (43.40 to 54.01)	0.137			
Working memory (reaction time)	CON	1013.14 (950.99 to 1075.29)	946.66 (893.18 to 1000.15)	0.089	−17.36 (−123.62 to 88.89)	0.744	0.0
	TWM-E	1013.85 (955.00 to 1073.70)	930.01 (878.55 to 981.46)	0.026			

* Log transformations have occurred.
